# Retention-Aware DRAM Auto-Refresh Scheme for Energy and Performance Efficiency

**DOI:** 10.3390/mi10090590

**Published:** 2019-09-08

**Authors:** Wei-Kai Cheng, Po-Yuan Shen, Xin-Lun Li

**Affiliations:** Department of Information and Computer Engineering, Chung Yuan Christian University, Taoyuan 32023, Taiwan

**Keywords:** DRAM refresh, retention time, refresh interval, refresh cycle time, auto-refresh

## Abstract

Dynamic random access memory (DRAM) circuits require periodic refresh operations to prevent data loss. As DRAM density increases, DRAM refresh overhead is even worse due to the increase of the refresh cycle time. However, because of few the cells in memory that have lower retention time, DRAM has to raise the refresh frequency to keep the data integrity, and hence produce unnecessary refreshes for the other normal cells, which results in a large refresh energy and performance delay of memory access. In this paper, we propose an integration scheme for DRAM refresh based on the retention-aware auto-refresh (RAAR) method and 2x granularity auto-refresh simultaneously. We also explain the corresponding modification need on memory controllers to support the proposed integration refresh scheme. With the given profile of weak cells distribution in memory banks, our integration scheme can choose the most appropriate refresh technique in each refresh time. Experimental results on different refresh cycle times show that the retention-aware refresh scheme can properly improve the system performance and have a great reduction in refresh energy. Especially when the number of weak cells increased due to the thermal effect of 3D-stacked architecture, our methodology still keeps the same performance and energy efficiency.

## 1. Introduction

Dynamic random access memory (DRAM) is widely used in electronic devices. DRAM has a high performance and low cost, and a DRAM cell is composed of an access transistor and one leaky capacitor, each DRAM cell stores one-bit of data as an electrical charge in a capacitor. DRAM cells leak charge over time, causing stored data lost. To prevent this situation, DRAM cells require recharge periodically. These recharge operations are also known as DRAM refresh.

There are two important parameters for DRAM refresh, refresh interval (tREFI) and refresh cycle time (tRFC). The memory controller sends a refresh command to DRAM devices every tREFI (7.8 us as usual), and the duration of a refresh command is referred to as tRFC. However, the value of tRFC worsens when the DRAM capacity increases. As described in Table 1 [1], we calculate refresh overhead by tRFC/tREFI. This value is 890 ns/7.8 us in a 32 Gb DRAM, it takes about 12.49% of refresh time to refresh all its cells. The larger the memory size, it will have more serious performance degradation and energy consumption.

In the JEDEC standard [2], DRAM cells are refreshed every 64 ms at normal temperature (<85 °C) and 32 ms at high temperature (>85 °C). However, most of the DRAM cells have a longer retention time (tRET) than 64 ms. Research [3] indicated that in a 32 GB DDR3 DRAM device, only about 30 cells’ retention time is less than 128 ms, and only about 1000 cells require a refresh interval shorter than 256 ms. We define these low retention cells as weak cells, and a memory row that contains weak cells is defined as a weak row. It is obvious that a refresh of all rows every 64 ms is unnecessary since only a few memory rows are weak rows. Because of the few low retention time cells, this fixed minimum refresh interval scheme leads to a large amount of unnecessary refresh power consumption and also degrades the service efficiency of memory requests [3].

Because of the restriction of cell retention time, except the fixed refresh interval problem, another cause of a degrading memory access performance comes from the fact that DRAM refresh operation must have higher priority than memory request service. While in the refresh period, the memory request service of the rank being refreshed is forced to wait. In general, the memory controller issues an auto-refresh command at a fixed interval. It cannot ensure that the memory refresh and memory access request does not collide. Commodity DRAM device performs refresh operations at rank-level and leads to the idling of all banks in the rank during the refresh period.

There has been some research on reducing the overhead of DRAM by retention-aware refresh or refresh scheduling [3,4,5,6,7,8,9,10,11,12]. Research [4] proposed a RAAR technique to reduce refresh energy and performance degradation, only part of refresh bundles need a refresh in each refresh interval, while other refresh bundles can skip at least 50% of refresh operations. Although this methodology could reduce a lot of redundant refreshes, because it chose a unique refresh scheme all over the RAAR period, it still wastes quite a number of refresh time on normal cells. Research [5] improved this phenomenon by integrating all the RAAR refresh schemes and chose the optimal refresh scheme for different refresh bundles to minimize refresh time and refresh energy. Research [6] integrated the RAAR scheme with the bank reordering technique to gather up weak rows in adjacent banks, and hence less refresh bundles need refresh in each refresh interval. As observed in research [7], a refreshing bundle that contains weak rows still contain lots of normal cells even if we choose the optimal refresh scheme for it. Therefore, research [8] proposed an integration scheme to integrate RAAR and built-in self-repair (BISR) technique, selected weak rows are repaired with the BISR technique to minimize refresh overhead of refresh bundles.

Other related works to improve refresh efficiency are introduced below. Research [3] grouped DRAM rows into retention time bins and applied a different refresh rate to each bin. Research [7] presented a DRAM refresh technique that simultaneously leveraged bank-level and subarray-level concurrency to reduce the overhead of distributed refresh operations in the hybrid memory cube. A bundle of DRAM rows in a refresh operation was composed of two subgroups, both subgroups of DRAM rows were refreshed concurrently during a refresh command to reduce refresh cycle time. Research [9] proposed to issue per-bank refreshes to idle banks in an out-of-order manner instead of round-robin order. Research [10] applied a refresh interval longer than the conventional refresh interval (64 ms), and memory controller issued read operations to the weak rows every required refresh interval in order to retain the data in weak rows. Research [11] proposed an extension to the existing DRAM control register access protocol to include the internal refresh counter and introduced a new dummy refresh command hat skipped refresh operations and simply incremented the internal counter. Research [12] proposed a set of techniques to coordinate the scheduling of low power mode transitions and refresh commands such that most of the required refreshes were scheduled in the low power self-refresh mode. Research [13] mitigated refresh overhead by a caching scheme based on the fact that ranks in the same channel were refreshed in a staggered manner.

In summary of the related works that addressed the RAAR technique, although research [5] had proposed an integration refresh scheme, it did not integrate the 2x granularity auto-refresh and hence the performance improvement was less than 10%. Research [6] and research [8] also reveal the fact that although they integrated RAAR scheme with bank ordering and BISR technique, respectively, there was still very little performance improvement achieved. All these were because that weak cells only occupy a low percentage of all memory cells, if the 2x granularity was not integrated into the refresh scheme, all other optimization techniques can only obtain limited reduction on refresh overhead. For other related RAAR related techniques [3,9,10,11], they also did not take into account the 2x granularity issue in their works. Based on these observations, in this paper, we propose an integration scheme for DRAM refresh based on the retention-aware auto-refresh (RAAR) method and 2x granularity auto-refresh simultaneously, and the corresponding memory controller needs to support the proposed integration refresh scheme. With a given profile of weak cells distribution in memory banks, our integration scheme can choose the most appropriate refresh technique in each refresh time.

The rest of this paper is organized as follows. Section 2 shows the background of DRAM organization, refresh interval, refresh cycle time, and refresh command. Section 3 illustrates the motivation of our integration refresh scheme. In Section 4, we propose the 2x granularity auto-refresh, integration refresh scheme, and corresponding memory controller. Section 5 shows the evaluation results of our methodology on different weak row percentage and refresh cycle time. Finally, we draw concluding remarks in Section 6.

## 2. Background

**Figure 1:**
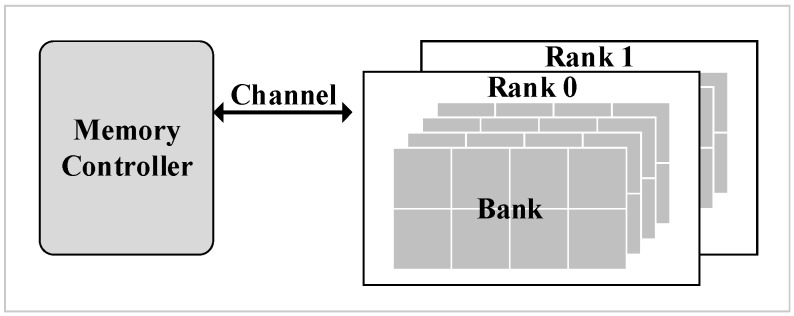
Dynamic random access memory (DRAM) hierarchical organization.

The modern DRAM system is a hierarchical organization [9]. The hierarchies from high to low levels are rank and bank as shown in Figure 1. Each channel connects one or more ranks to a memory controller. Furthermore, banks are an array structure containing rows and columns, in which each cell stores one bit of data. However, cells leak charge over time which causes data loss.

**Figure 2:**
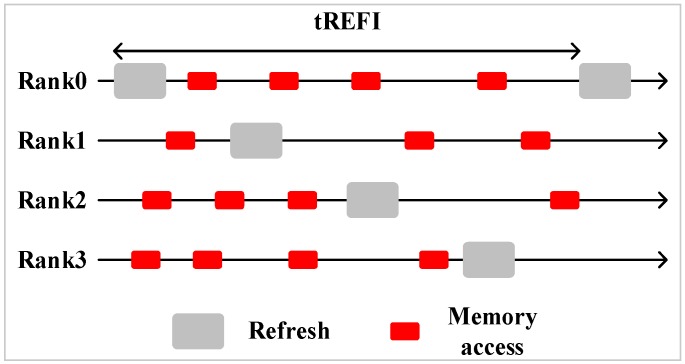
Staggered auto-refresh in a four-rank system.

Multiple ranks sharing one channel is common in a modern memory system. In a multi-rank system, all ranks’ refresh operations are staggered, there is no overlap among each rank’s refresh. The advantage of a multi-rank memory system is that while one rank is refreshing, the other ranks can still serve memory requests. Figure 2 shows a staggered refresh in a four-rank memory system.

There are three major parts that contribute to the power consumption of DRAM, background power, active power, and refresh power. Background power comes from peripheral circuitry energy consumption. Low power mode disables some peripheral circuitry to reduce background power consumption. Active power is only consumed while servicing a memory request. To service a memory request, the target row must be first activated and precharged. Since a DRAM cell is composed of an access transistor and a leaky capacitor, the DRAM is required to be refreshed periodically and thereby dissipating its refresh power. According to our evaluation, refresh power accounts up to 25–27% of the total energy consumption in a 32 GB DRAM device, and this percentage will even increase as the DRAM size increased in the future [3]. Therefore, refresh power reduction becomes a crucial issue in the future.

**Figure 3:**
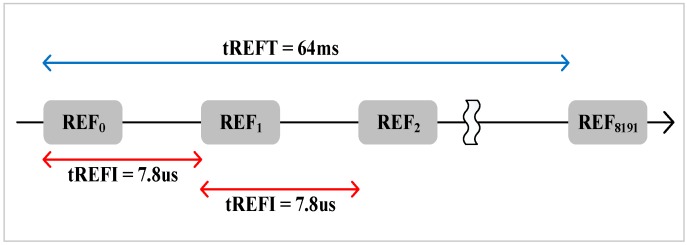
All rows are refreshed by 8K refresh commands.

Refreshing all rows at the same time leads to long refresh latency. Therefore, to avoid long refresh latency, the total rows in a bank are divided into 8K groups [13]. It is evenly distributing the refresh latency among runtime. That is to say, 8K auto-refresh commands are issued to refresh a subset of rows in 64 ms interval. As the example shown in Figure 3, 8K auto-refresh commands are issued every 64 ms/8K = 7.8 us.

**Figure 4:**
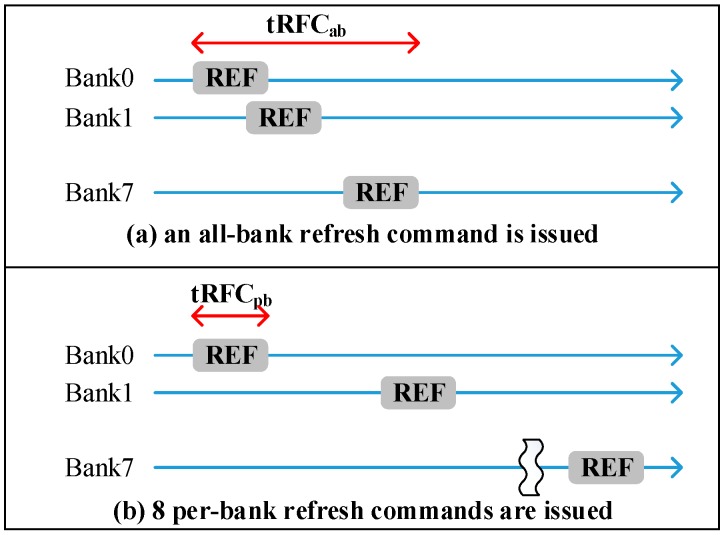
Comparisons of an all-bank refresh and per-bank refresh.

Commodity DRAM device supports all-bank auto-refresh. All banks are unavailable for tRFC cycle times while all-bank auto-refresh commands are issued. In contrast, per-bank auto-refresh [14] is also a supported refresh technique in a standard DRAM device. Per-bank refresh divides one all-bank refresh into eight per-bank refreshes. Per-bank refreshes only refresh one bank at a time. In other words, only one bank is idle for refresh and the other banks can still serve memory requests. The advantage of per-bank refresh is that it is more efficient for one bank refresh than all-bank refresh due to shorter tRFC. Figure 4 illustrates the comparisons of all-bank refresh and per-bank refresh, tRFCpb is about 2.3x shorter than tRFCab.

**Figure 5:**
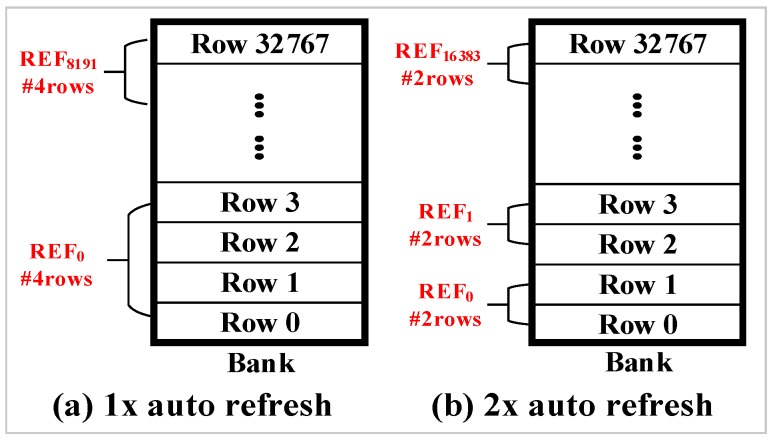
Illustrations of (**a**) 1x and (**b**) 2x auto-refresh.

As described previously, the total number of rows in a bank is divided into 8K groups for refresh operations. In addition to dividing rows into 8K groups, JEDEC DDR4 standard [15] also support 2x granularity auto-refresh. The 2x granularity auto-refresh scheme divides the total number of rows in a bank into 16K groups. Therefore, only half of the rows are refreshed per auto-refresh command. Figure 5a shows a bank composed of 32K rows. The 32K rows are divided into 8K groups by default. Therefore, four rows are refreshed in a bank per auto-refresh command. Figure 5b shows an example of 2x granularity auto-refresh. It divides the total number of rows in a bank into 16K groups and only two rows are refreshed per refresh operation.

## 3. Motivation and Example

In a DRAM refresh, a refresh command includes 8192 refresh times in 64 ms of a refresh interval. Although RAAR reduces a large number of unnecessary refreshes, some banks that do not contain weak rows still have to be refreshed because of all-bank refresh mode. Also, although 2x granularity reduces refresh interval to one half of that in 1x granularity, its refresh cycle time exceeds one half of that in 1x granularity. For example of the 8 Gb DRAM, for 1x granularity its refresh interval is 7.8 us and refresh cycle time is 350 ns, and for 2x granularity its refresh interval is 3.9us and refresh cycle time is 260 ns. Two 2x granularity refresh requires 520 ns while one 1x granularity refresh only require 350 ns. Therefore, a retention-aware refresh scheme to corporate with 2x granularity refresh is necessary.

For the RAAR technique proposed by research [4], only refresh bundles that contain weak rows need a refresh in each refresh interval. However, weak rows distribution between refresh bundles may differ dramatically. If the same refresh scheme is used all over the RAAR period, there still will be quite number of refresh time wasted due to extra normal cells refreshing or bad weak rows refreshing sequentially.

**Figure 6:**
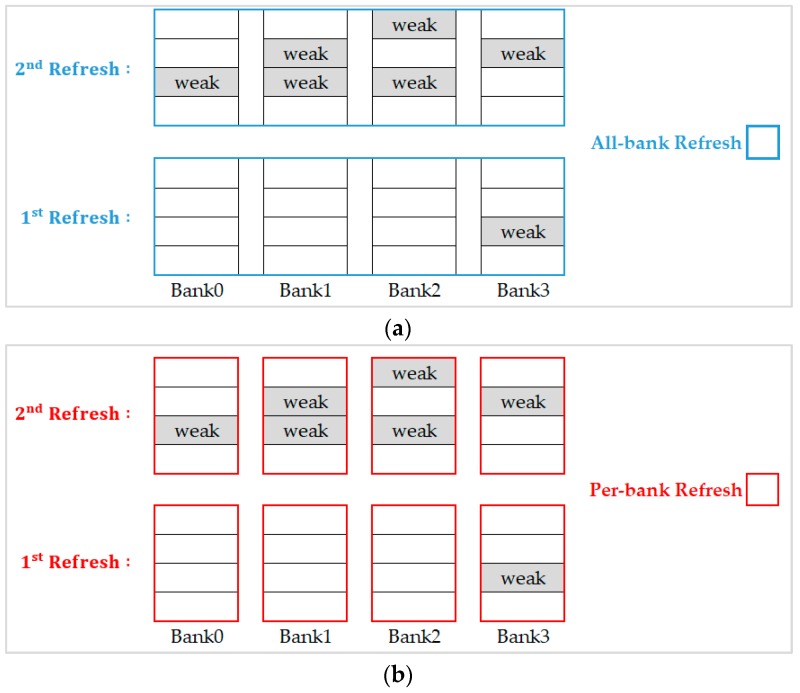
(**a**) All-bank refresh to refresh weak row; (**b**) per-bank refresh to refresh weak row.

For the example illustrated in Figure 6, we executed refresh commands with the same refresh scheme all over the refresh interval (refresh 8192 times in 64 ms). Figure 6a shows the results when choosing the all-bank refresh scheme, we see that it performs badly in the first refresh because of three unnecessary banks to be refreshed simultaneously. However, this scheme performs well for the second refresh since all banks contain weak rows. On the other hand, when we choose the per-bank refresh scheme as shown in Figure 6b, although it is appropriate for the first refresh, it needs to refresh all the banks sequentially and causes a lot of waste of refresh time and refresh energy when it starts on the second refresh.

In research [5], in addition to all-bank refresh and per-bank refresh, two new fine-granularity refresh schemes, namely half-bank refresh, and quarter-bank refresh were proposed to further reduce unnecessary refresh. Although this integration refresh scheme combined both the advantage of all-bank refresh and per-bank refresh, provided fine-granularity refresh modes selection to resolve the problem of too much unnecessary refresh in all-bank refresh and high refresh overhead in per-bank refresh, it did not integrate the 2x granularity auto-refresh and hence the performance improvement was less than 10%. As described at the beginning of this section, replacing the 1x granularity auto-refresh with 2x granularity auto-refresh directly without co-operating with the retention-aware refresh scheme is useless. In this paper, we propose a methodology to integrate 2x granularity auto-refresh and the retention-aware refresh scheme which can have significant performance improvement as shown in experimental results.

## 4. Methodology

In this section, we describe our approach for retention-aware refresh optimization, including fine granularity RAAR, integration refresh scheme, and the corresponding memory controller to support the proposed integration refresh scheme.

### 4.1. 2x Granularity Retention-Aware Auto-Refresh (RAAR)

Since there are large amounts of rows that are unnecessary to be refreshed in every minimal retention time refresh interval (tREFT) (64 ms as usual), RAAR profiles the DRAM rows’ retention time and tags the weak rows, non-weak rows were not refreshed every tREFI. The key idea of RAAR is to only refresh weak rows in a high frequency and skip refreshing non-weak rows. Therefore, refreshing fewer rows per auto-refresh command is better for RAAR. RAAR adopts the default auto-refresh and 2x granularity auto-refresh to reduce unnecessary rows being refreshed. Furthermore, to skip refresh non-weak rows, we accessed and increased the value of the internal refresh counter without refreshing rows.

**Figure 7:**
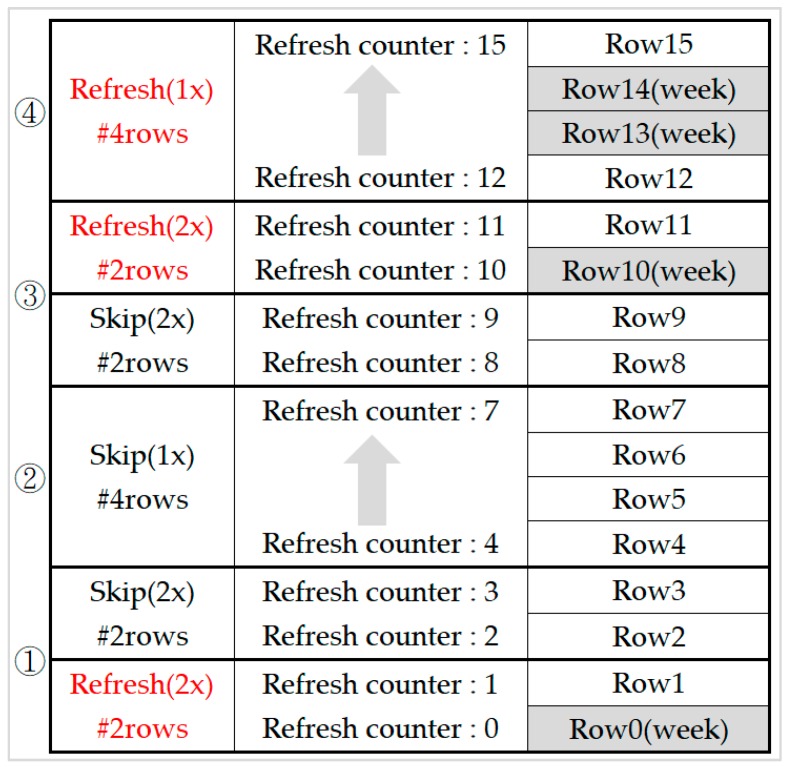
Retention-aware auto-refresh.

Figure 7 illustrates how RAAR works. There are sixteen rows in a bank and only four of them are weak rows (row0, 10, 13, 14). We assumed a 1x auto-refresh command that refreshes four rows. At first refresh (1), it refreshes row0–row3. However, only row0 is a weak row. Therefore, we use two 2x auto-refresh commands to refresh these four rows. Actually, only the first 2x auto-refresh command refresh row0 and row1 because of weak row0. The second 2x auto-refresh command only modifies the refresh counter from two to three and does not incur any refresh overhead. As per the second refresh (2) shown in Figure 7, there is no weak row in row4–row7. We only increased the refresh counter by 1x auto-refresh without any refresh overhead. The condition of the third refresh (3) was similar to the first refresh (1), only one weak row exits. Two 2x auto-refresh commands were issued but actually only refresh row10 and row11. In the fourth refresh (4), both the first half and second half contain a weak row. So, we refreshed these four rows as normal by one 1x auto-refresh. In this example, RAAR only refreshed eight rows rather than refreshing all sixteen rows.

### 4.2. Integration Refresh Scheme

**Figure 8:**
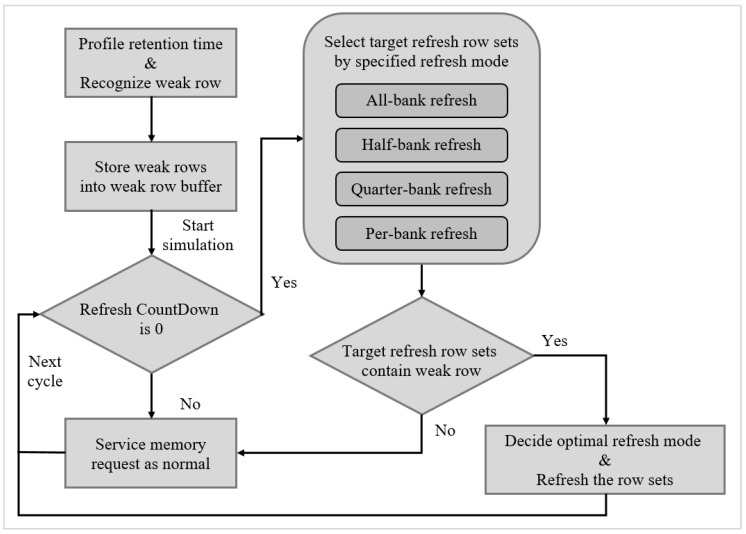
Proposed program flow.

Figure 8 shows the program flow of our integration refresh scheme. After the testing and measurement, we firstly produce the profile of retention time under different thermal environment and store the information of weak rows into a weak row buffer. A 64 ms refresh counter was used to issue the refresh command for weak rows. For each 64 ms interval, we selected target row sets by a specified refresh mode. When the target refresh row sets contained weak rows, we then decided the optimal refresh mode and refreshed the row sets. Otherwise, the memory controller would provide service for memory requests as normal.

**Figure 9:**
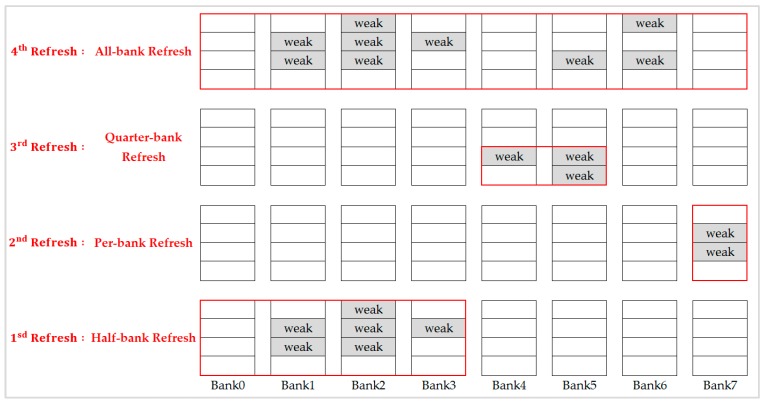
Integration refresh scheme in each refresh.

The decision of refresh mode for a row set is based on its weak rows distribution, the respective tRFC of each refresh scheme, and the 2x granularity refresh method in JEDEC DDR4 standard [15]. Table 2 shows the tRFC ratio of different refresh modes used in our integration refresh scheme. Because per-bank refresh is already a fine granularity refresh scheme, 2x granularity auto-refresh is not applied to it. We calculated the refresh overhead of each refresh scheme for the target row set and chose the most appropriate refresh mode. Figure 9 shows an example of integration refresh scheme to mitigate an unnecessary refresh. In a refresh of the first-row set, we chose one half-bank refresh, which was better than a two quarter-bank refresh or three per-bank refresh. In the refresh of the second-row set, one per-bank refresh was the best one. In refresh of the third-row set, one quarter-bank refresh was better than two per-bank refresh. And in refresh of the fourth-row set, one all-bank refresh was the best, which was better than two half-bank refresh, four quarter-bank refresh, or five per-bank refresh.

### 4.3. Memory Controller

**Figure 10:**
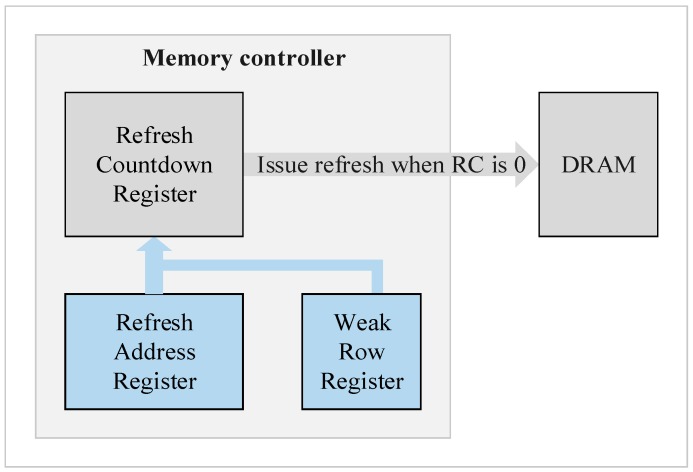
Modified memory controller.

To accomplish the integration refresh scheme, a little modification on the memory controller is required, which is also the piece of the overhead of our proposal. Figure 10 shows the modification of the memory controller to fit our integration refresh scheme. In the original memory controller, there is a refresh countdown register to issue a refresh command according to the refresh period. When the register counts down to zero, the memory controller issues a refresh command. However, the memory controller cannot specify the rows to be refreshed, which is determined by the row counter inside the DRAM. Therefore, we added two extra registers for the need of our integration refresh scheme—a refresh address register to monitor the refresh rows and a weak row register to record weak rows. Since the profile of weak cells distribution in memory banks are obtained after the testing and measurement analysis of the memory wafer, we do not need to detect weak rows on the fly, and only address the weak rows that are recorded in the weak row register, no bitmap for all rows nor a counter for each row is necessary. In addition, since the profiles are produced after the wafer measurement, we either do not need to change refresh scheme on the fly, as refresh commands for different refresh bundles are prepared based on our integration refresh scheme in advance.

**Figure 11:**
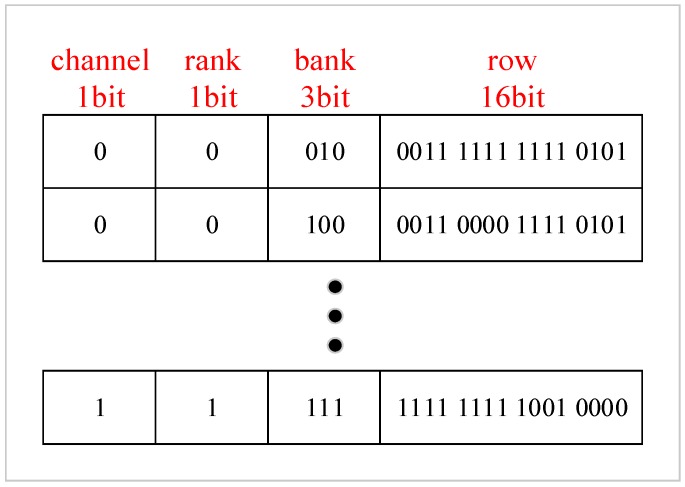
Organization of weak row register.

Figure 11 shows the organization of a weak row register. For the example of MT41K1G8 [1], the memory architecture has two channels and two ranks, each rank has eight banks, and each bank has 64K rows. When the memory architecture is changed, bit organization of weak row register is also modified accordingly. Basically, the weak row percentage is less than 0.5% in normal, and even in a worse environment this percentage is less than 1% for almost all operating conditions [3]. This means that less than 13440 bit (640 register entries) space is required for 32 Gb DRAM in standard conditions, and 67200 bit (3200 register entries) space is required for the extreme case of a 5% weak row percentage which has probability near zero. In addition, since the weak row profile is produced after wafer measurement and we do not need to change it on the fly, we can use Mask ROM or Nor Flash instead of logic registers to record these data, which greatly reduce the overhead. Therefore, the overhead is about 1 KB of Mask ROM or Nor Flash for most operating conditions, and less than 8 KB of Mask ROM or Nor Flash in the extreme case.

**Figure 12:**
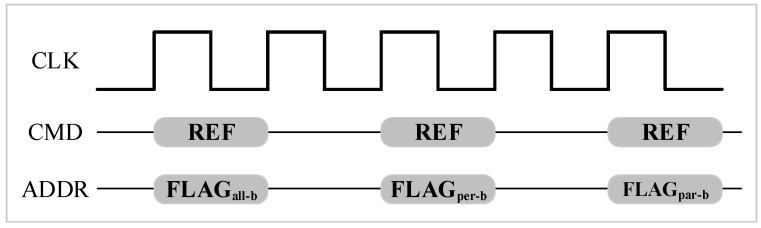
Refresh commands with different flags.

Figure 12 shows the refresh modes implementation. In addition to the refresh command, we added refresh mode flags on the ADDR field to identify which refresh mode was performed in the refresh. Also, the command decoder inside DRAM needs the corresponding modification to identify refresh mode, and a register to record the refresh banks. Because we do not need to detect weak rows on the fly, refresh commands for different refresh bundles were prepared in advance and sent to the memory controller by the CPU based on our integration refresh scheme, so only very little overhead on memory controller is required.

## 5. Experimental Results

**Figure 13:**
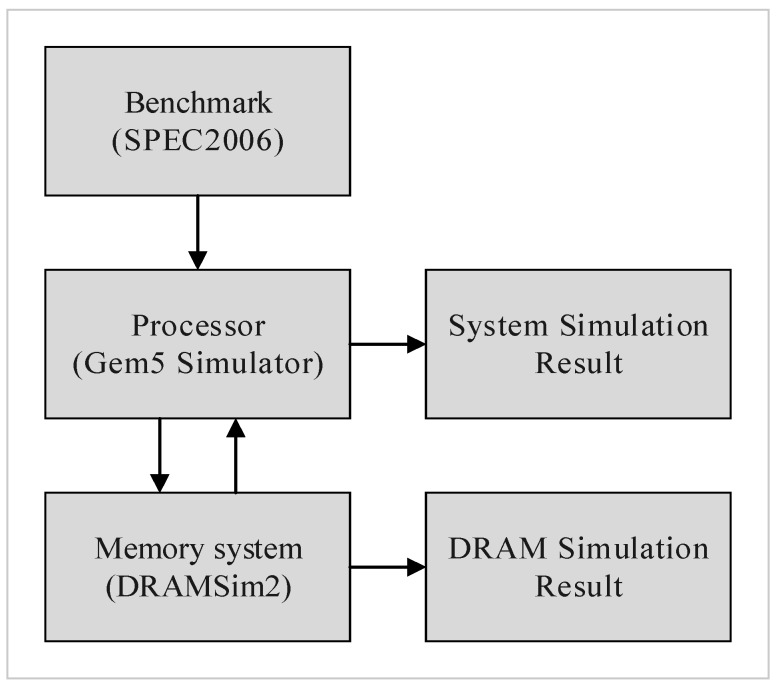
Evaluation flow.

We integrated Gem5 [16] and DRAMSim2 [17] as our simulation environment, Figure 13 shows our evaluation flow. Gem5 provided the execution trace and system performance in terms of instructions-per-clock (IPC), and DRAMSim2 provided a detailed active power, refresh power, and background power. We applied eight evaluation benchmarks from SPEC 2006, including four memory-bound benchmarks (mcf, lib, sjeng, cact) and four CPU-bound benchmarks (cal, xal, sop, bzip2). We used the default CPU and memory architecture settings of Gem5 and DRAMSim2, DRAM specification was based on the Micron MT41K1G8 datasheet [1], and the system configurations are shown in Table 3. Our experiments only classify memory cells into two categories: weak cells and normal cells, because more levels of weakness just increase the complexity of the refresh control but almost has no further improvement in reducing refresh overhead. To compare the effect of different refresh cycle times on different DRAM capacities, Table 3 lists the tRFC of all-bank auto-refresh for 16 Gb and 32 Gb DRAM capacities, while tRFC of per-bank auto-refresh, half-bank auto-refresh, and quarter-bank auto-refresh are calculated as the ratio setting in Table 2. We take the default 1x granularity all-bank auto-refresh as the evaluation baseline, and compare four refresh schemes including 2x granularity all-bank auto-refresh, 1x granularity per-bank auto-refresh, 2x granularity half-bank auto-refresh (partial bank refresh), and the proposed 2x granularity integration refresh scheme. In a normal operating environment, the weak row percentage is about 1% or less than 1% [3]. Since 3D-stacked architecture is the future trend, we monitor this operating environment in which weak rows increased due to thermal effect on cells’ retention time. For this reason, we evaluate our 2x granularity integration refresh scheme under higher weak row percentage (1–5%) with random weak rows distribution.

**Figure 14:**
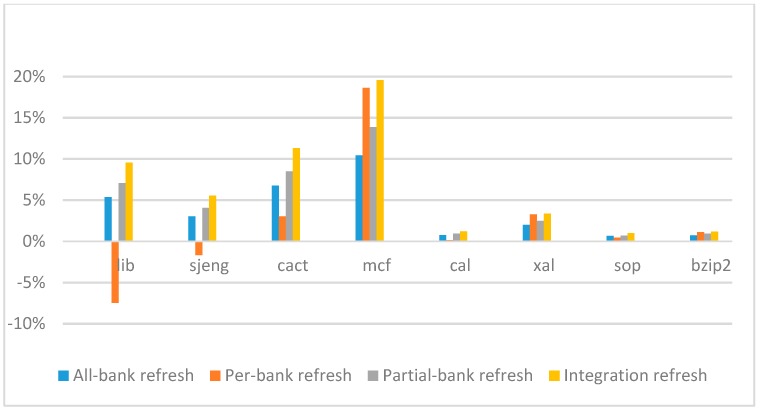
Instructions-per-clock (IPC) improvement normalized to 1x granularity baseline (tRFC = 530 ns, weak row = 1%).

**Figure 15:**
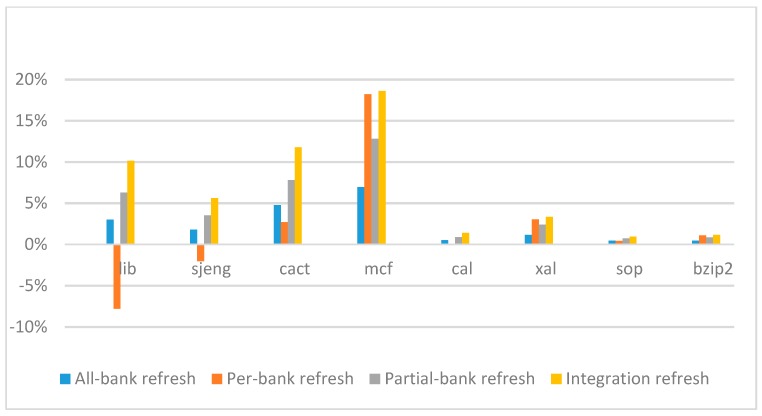
IPC improvement normalized to 1x granularity baseline (tRFC = 530 ns, weak row = 2%).

**Figure 16:**
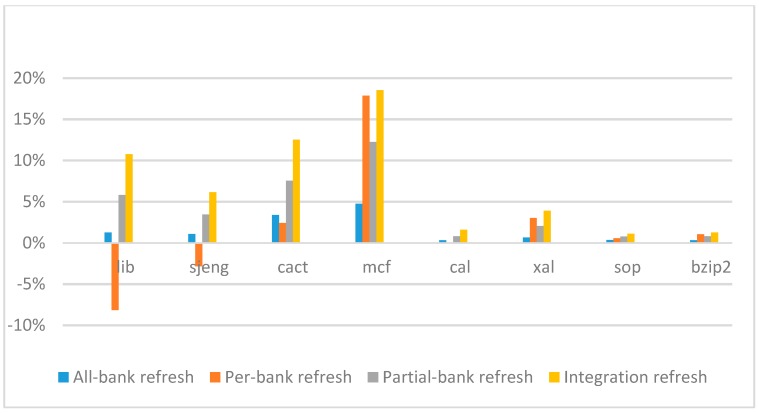
IPC improvement normalized to 1x granularity baseline (tRFC = 530 ns, weak row = 3%).

**Figure 17:**
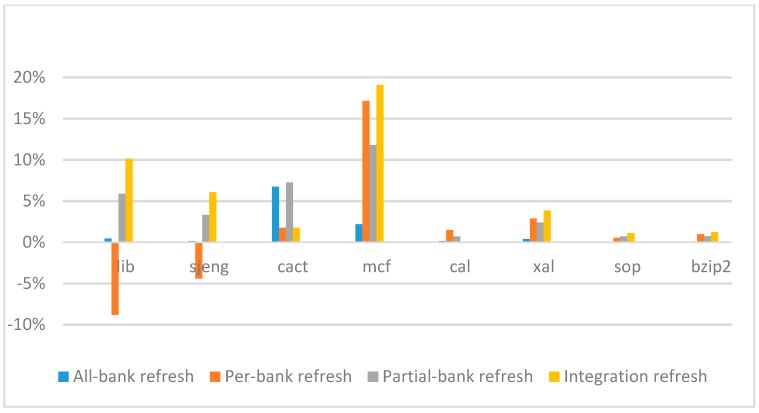
IPC improvement normalized to 1x granularity baseline (tRFC = 530 ns, weak row = 5%).

Figure 14, Figure 15, Figure 16 and Figure 17 show the IPC improvement of the four 2x granularity refresh schemes (except per-bank refresh) over the default 1x granularity all-bank refresh baseline on the 16 Gb DRAM with tRFC_ab_ equal to 530 ns, and weak row percentage is 1%, 2%, 3%, and 5%, respectively. For the four memory-bound benchmarks, per-bank refresh scheme has worse results on the lib and sjeng benchmarks in comparison with the baseline, while our integration refresh scheme all get the best results except for the cat benchmark when weak row percentage is 5%. On the mcf benchmark, integration refresh scheme achieves near 20% of IPC improvement, and at least achieves over 5% of improvement on the sjeng benchmark. For the four CPU-bound benchmarks, our approach still has the best results except for the cact and cal benchmarks when the weak row percentage is 5%, but all get less than 5% of improvement.

**Figure 18:**
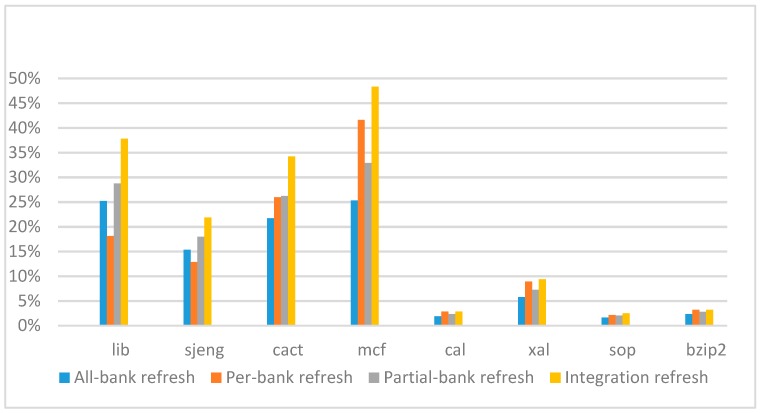
IPC improvement normalized to 1x granularity baseline (tRFC = 890 ns, weak row = 1%).

**Figure 19:**
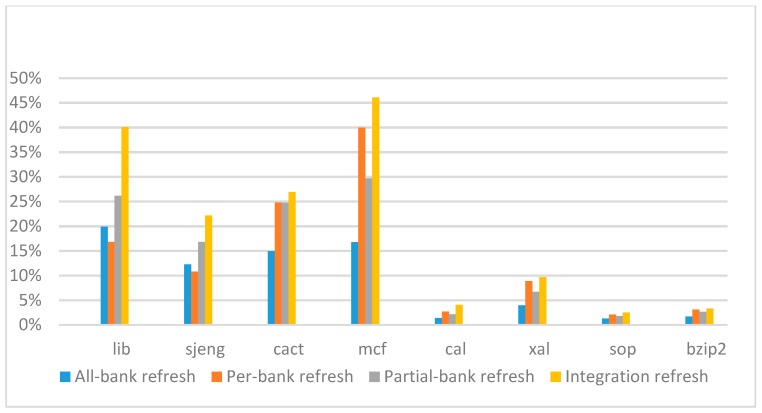
IPC improvement normalized to 1x granularity baseline (tRFC=890 ns, weak row = 2%).

**Figure 20:**
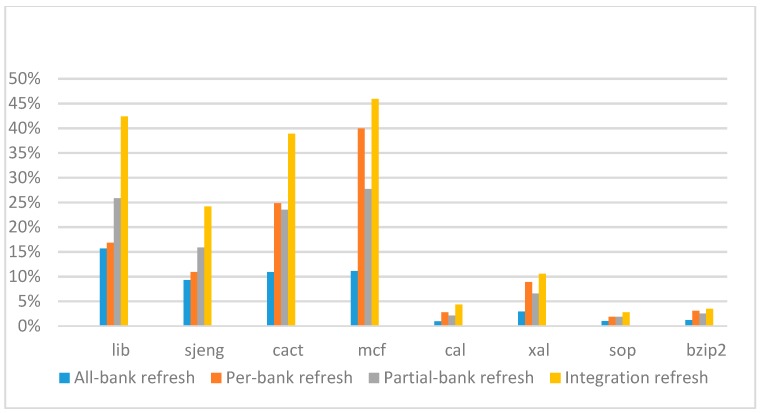
IPC improvement normalized to 1x granularity baseline (tRFC = 890 ns, weak row = 3%).

**Figure 21:**
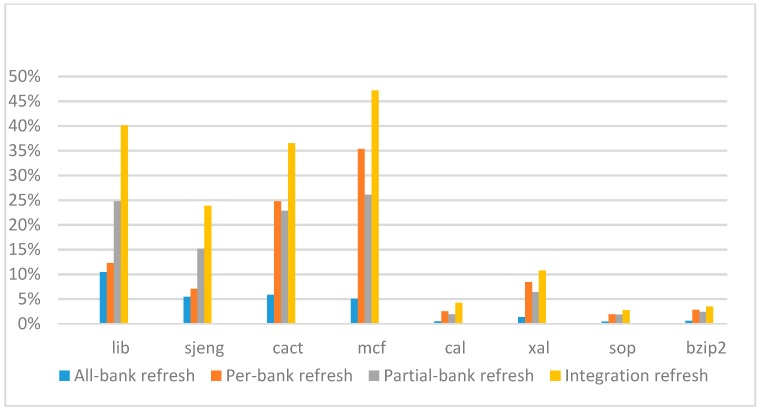
IPC improvement normalized to 1x granularity baseline (tRFC = 890 ns, weak row = 5%).

Figure 18, Figure 19, Figure 20 and Figure 21 show the IPC improvement of the four 2x granularity refresh schemes (except per-bank refresh) over the default 1x granularity all-bank refresh baseline on the 32 Gb DRAM with tRFC_ab_ equal to 890 ns, and weak row percentage is 1%, 2%, 3%, and 5%, respectively. For the four memory-bound benchmarks, our integration refresh scheme all get the best results. On the mcf benchmark, integration refresh scheme achieves over 45% of IPC improvement, and at least achieves over 20% of improvement on the *sjeng* benchmark. While for the four CPU-bound benchmarks, our approach still has the best result especially on the high weak row percentage environment, and near the per-bank refresh scheme on the low weak row percentage environment. Because of less memory access, the impact of refresh operations on IPC is degraded, all four refresh schemes achieve much less of IPC improvement on CPU-bound benchmarks, but our approach still has near 10% of improvement.

From the experimental results on different refresh cycle times, we see that as DRAM size increased, the 2x granularity integration refresh scheme has an even more significant effect on the performance improvement, especially for memory-bound applications. Comparing these four 2x granularity refresh schemes, our integration refresh scheme almost has no performance degradation on all the benchmarks except cact when the weak row percentage increased from 1% to 5%. However, all three other refresh schemes have performance degradation as the weak row percentage increased, especially for the 2x granularity all-bank refresh scheme. Also, the performance of per-bank refresh scheme is not stable in comparison with other refresh schemes, it has even worse results than the baseline on two benchmarks in the case of smaller refresh cycle time. The reason that per-bank refresh performed badly on the lib and sjeng benchmarks is due to various memory access patterns on different benchmarks. Per-bank refresh scheme performs better on disjoint memory location accessed patterns, while the lib and sjeng benchmarks have more closed memory location accessed patterns. This shows that our approach has the strongest ability to against thermal variation and refresh cycle time variation, and has the best performance improvement.

**Figure 22:**
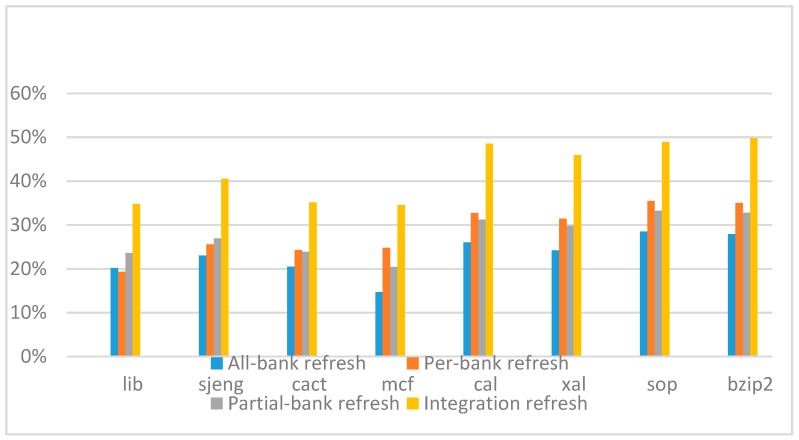
Energy reduction normalized to 1x granularity baseline (tRFC = 530 ns, weak row = 1%).

**Figure 23:**
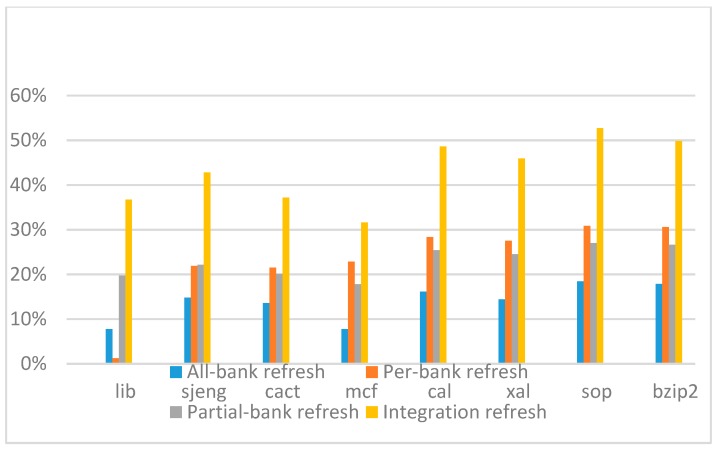
Energy reduction normalized to 1x granularity baseline (tRFC = 530 ns, weak row = 2%).

**Figure 24:**
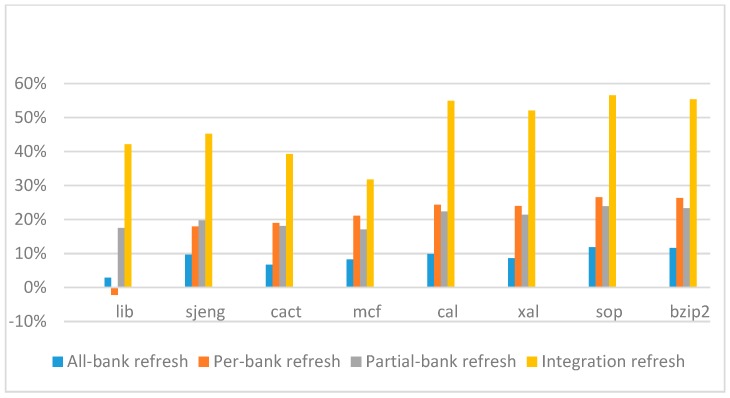
Energy reduction normalized to 1x granularity baseline (tRFC = 530 ns, weak row = 3%).

**Figure 25:**
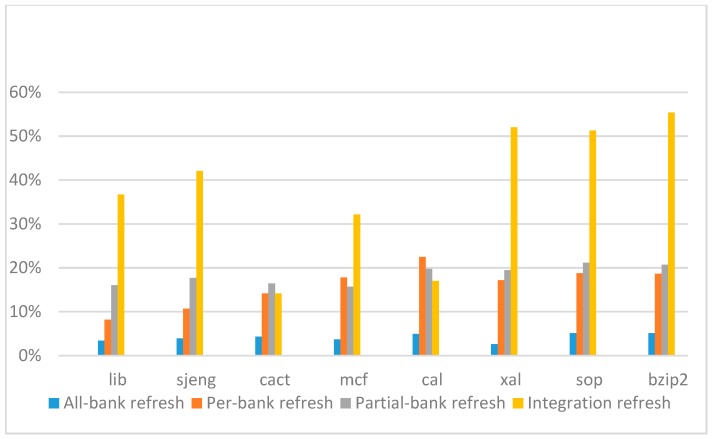
Energy reduction normalized to 1x granularity baseline (tRFC = 530 ns, weak row = 5%).

Figure 22, Figure 23, Figure 24 and Figure 25 show the refresh energy reduction of the four 2x granularity refresh schemes (except per-bank refresh) over the default 1x granularity all-bank refresh baseline on the 16 Gb DRAM with tRFC equal to 530 ns, and the weak row percentage is 1%, 2%, 3%, and 5%, respectively. Different from the experiments on performance evaluation, both memory-bound benchmarks and CPU-bound benchmarks gained an obvious improvement in refresh energy reduction, CPU-bound benchmarks even obtained more reduction than memory-bound benchmarks. Also, our 2x granularity integration refresh scheme all get the best results except for the cact and cal benchmarks when the weak row percentage is 5%, and per-bank refresh scheme still performed badly for the lib and sjeng benchmarks.

**Figure 26:**
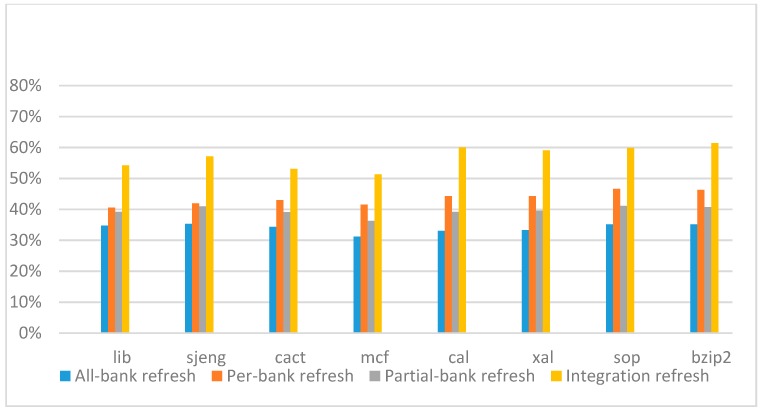
Energy reduction normalized to 1x granularity baseline (tRFC = 890 ns, weak row = 1%).

**Figure 27:**
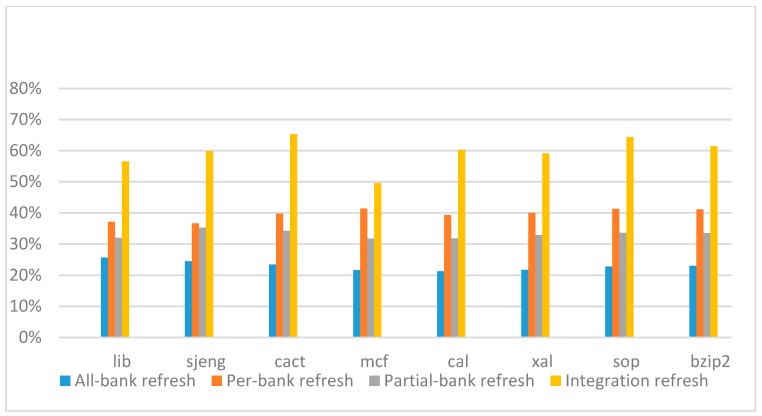
Energy reduction normalized to 1x granularity baseline (tRFC = 890 ns, weak row = 2%).

**Figure 28:**
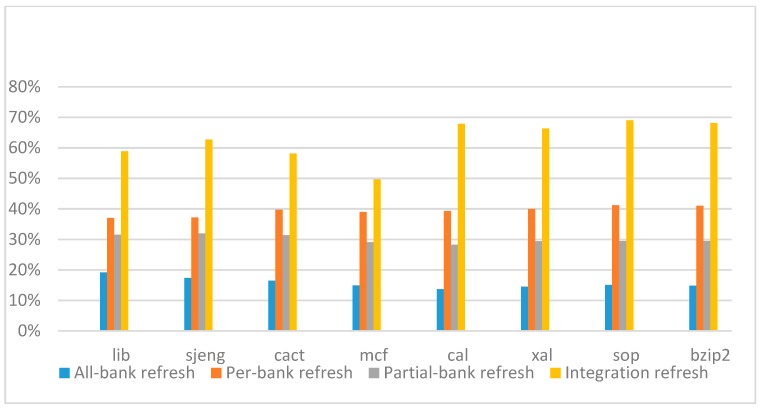
Energy reduction normalized to 1x granularity baseline (tRFC = 890 ns, weak row = 3%).

**Figure 29:**
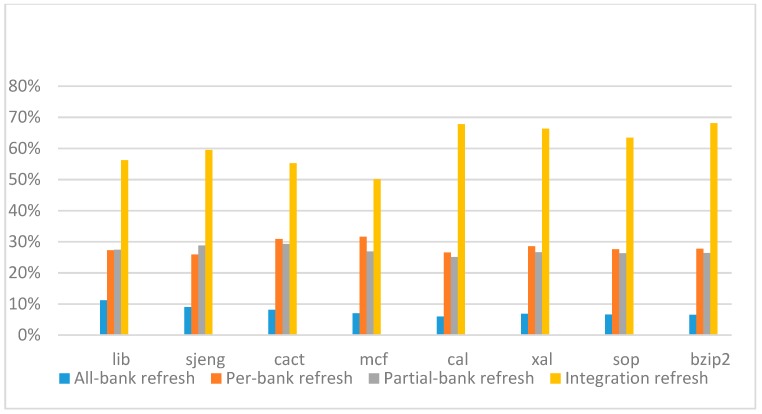
Energy reduction normalized to 1x granularity baseline (tRFC = 890 ns, weak row = 5%).

Figure 26, Figure 27, Figure 28 and Figure 29 show the refresh energy reduction of the four 2x granularity refresh schemes over the default 1x granularity all-bank refresh baseline on the 32 Gb DRAM with tRFC equal to 890 ns, and weak row percentage is 1%, 2%, 3%, and 5%, respectively. Comparing to the results of smaller refresh cycle time above, our integration refresh scheme further improves energy reduction for all benchmarks on all weak row percentage.

Comparing these four refresh schemes, our 2x granularity integration refresh scheme almost has no degradation on refresh energy reduction for all the benchmarks except *cact* when the weak row percentage increased from 1% to 5%. However, all three other refresh schemes have obvious degradation as the weak row percentage increased, especially for the 2x granularity all-bank refresh scheme, and as does the per-bank refresh scheme and 2x granularity half-bank refresh (partial-bank refresh) scheme when the weak row percentage increased to 5%. When DRAM size increased from 16 Gb to 32 Gb, per-bank refresh scheme has the largest improvement especially for the lib and sjeng benchmarks because of the finer granularity, and hence performed better than 2x granularity partial-bank refresh scheme on all benchmarks. Therefore, we further prove that our approach has the strongest ability to against thermal variation and refresh cycle time variation, and has the largest refresh energy reduction.

## 6. Conclusions

In this paper, we propose an integration scheme for DRAM refresh based on retention-aware auto-refresh and 2x granularity auto-refresh techniques simultaneously. Since the profile of weak cells distribution in memory banks are obtained after testing and measurement analysis of memory wafer, we do not need to detect weak rows on the fly. In addition, since the profile are produced after wafer measurement, we either do not need to change refresh scheme on the fly, refresh commands for different refresh bundles are prepared based on our integration refresh scheme in advance. Therefore, the hardware cost and control complexity on memory controller design to support this integration refresh scheme is acceptable and reasonable. Experimental results show that our retention-aware integration refresh scheme can properly improve the system performance and have a great reduction in DRAM refresh energy. From the results on different refresh cycle time, we see that as DRAM size increased, the 2x granularity integration refresh scheme has even more significant effect on the performance improvement, especially for memory-bound applications. Especially when the number of weak cells percentage increased due to thermal effect of 3D-stacked architecture, our approach has the strongest ability to against thermal variation and has the smallest refresh overhead. 

Most important of all, our integration refresh scheme has high extension flexibility. Even though for finer grain, for example, 4x granularity, our methodology can extend to suit different granularities easily, and the extra hardware cost and control complexity is acceptable and reasonable as described above. Also, as described previously, although 2x granularity reduces refresh interval to one half of that in 1x granularity, its refresh cycle time exceeds one half of that in 1x granularity. Therefore, not only finer grain individually can reduce refresh overhead, the integration with a refresh scheme like we propose is necessary. Since an increasing DRAM size and 3D-stacked architecture are the future directions, increased refresh cycle time and weak row percentage will be serious issues on the refresh problem, our methodology is indeed adequate and efficient to reduce refresh overhead. Moreover, there is serious thermal problem on the 3D-stacked architecture, the retention time of cells may vary as temperature changes in the CPU execution stage, and causes the number of weak cells and weak cells distribution to worsen. This phenomenon complicates the refresh problem, the overhead to detect weak rows and change refresh schemes on the fly is not a simple fix. Therefore, more measurement and analysis of cells weakness under different thermal environment is necessary. With more detailed weak cell profiles under thermal variation, the refresh scheme can take this issue into account and only needs to detect thermal variation, avoids to detect weak rows and change refresh schemes on the fly, which is important future work.

## Figures and Tables

**Table 1:** Refresh cycle time among different density.

Density	tRFC
1 Gb	110 ns
2 Gb	160 ns
4 Gb	260 ns
8 Gb	350 ns
16 Gb	530 ns
32 Gb	890 ns

**Table 2:** Refresh cycle time (tRFC) parameters definition and 2x granularity refresh check.

Refresh Mode	tRFC	2x Granularity
All-bank refresh	K	Y
Half-bank refresh	K/1.32	Y
Quarter-bank refresh	K/1.74	Y
Per-bank refresh	K/2.3	N

**Table 3:** System configurations.

Processor	1 Core, 3.2 GHz
L1 Cache	L1-I cache 128 KB, L1-D cache 128 KB
L2 Cache	L2 cache 4 MB
DRAM datasheet	Micron MT41K1G8, 32 Gb [1]
Memory frequency	800 MHz
Number of channels, ranks	1 channel, 4 ranks per channel
Number of banks, rows	8 banks per rank, 64K rows per bank
tRFC_ab_, weak row percentage	530 ns for 16 Gb, 890 ns for 32 Gb
	1%, 2%, 3%, 5% (Radom)
